# *Clostridioides difficile* exploits xanthine and uric acid as nutrients by utilizing a selenium-dependent catabolic pathway

**DOI:** 10.1128/spectrum.00844-24

**Published:** 2024-08-21

**Authors:** Michael A. Johnstone, William T. Self

**Affiliations:** 1 Burnett School of Biomedical Sciences, College of Medicine, University of Central Florida, Orlando, Florida, USA; College of New Jersey, Ewing, New Jersey, USA

**Keywords:** selenium-dependent molybdenum hydroxylase, selenium, *Clostridioides difficile*, purine metabolism, molybdoenzyme, uric acid, xanthine

## Abstract

Selenium is a trace element that plays critical roles in redox biology; it is typically incorporated into “selenoproteins” as the 21st amino acid selenocysteine. Additionally, selenium exists as a labile non-selenocysteine cofactor in a small subset of selenoproteins known as selenium-dependent molybdenum hydroxylases (SDMHs). In purinolytic clostridia, SDMHs are implicated in the degradation of hypoxanthine, xanthine, and uric acid for carbon and nitrogen. While SDMHs have been biochemically analyzed, the genes responsible for the insertion and maturation of the selenium cofactor lack characterization. In this study, we utilized the nosocomial pathogen *Clostridioides difficile* as a genetic model to begin characterizing this poorly understood selenium utilization pathway and its role in the catabolism of host-derived purines. We first observed that *C. difficile* could utilize hypoxanthine, xanthine, or uric acid to overcome a growth defect in a minimal medium devoid of glycine and threonine. However, strains lacking selenophosphate synthetase (*selD* mutants) still grew poorly in the presence of xanthine and uric acid, suggesting a selenium-dependent purinolytic process. Previous computational studies have identified *yqeB* and *yqeC* as potential candidates for cofactor maturation, so we subsequently deleted each gene using CRISPR-Cas9 technology. We surprisingly found that the growth of the Δ*yqeB* mutant in response to each purine was similar to the behavior of the *selD* mutants, while the Δ*yqeC* mutant exhibited no obvious phenotype. Our results suggest an important role for YqeB in selenium-dependent purine catabolism and also showcase *C. difficile* as an appropriate model organism to study the biological use of selenium.

**IMPORTANCE:**

The apparent modification of bacterial molybdenum hydroxylases with a catalytically essential selenium cofactor is the least understood mechanism of selenium incorporation. Selenium-dependent molybdenum hydroxylases play an important role in scavenging carbon and nitrogen from purines for purinolytic clostridia. Here, we used *Clostridioides difficile* as a genetic platform to begin dissecting the selenium cofactor trait and found genetic evidence for a selenium-dependent purinolytic pathway. The absence of *selD* or *yqeB*—a predicted genetic marker for the selenium cofactor trait—resulted in impaired growth on xanthine and uric acid, known substrates for selenium-dependent molybdenum hydroxylases. Our findings provide a genetic foundation for future research of this pathway and suggest a novel metabolic strategy for *C. difficile* to scavenge host-derived purines from the gut.

## INTRODUCTION

**Fig 1:**
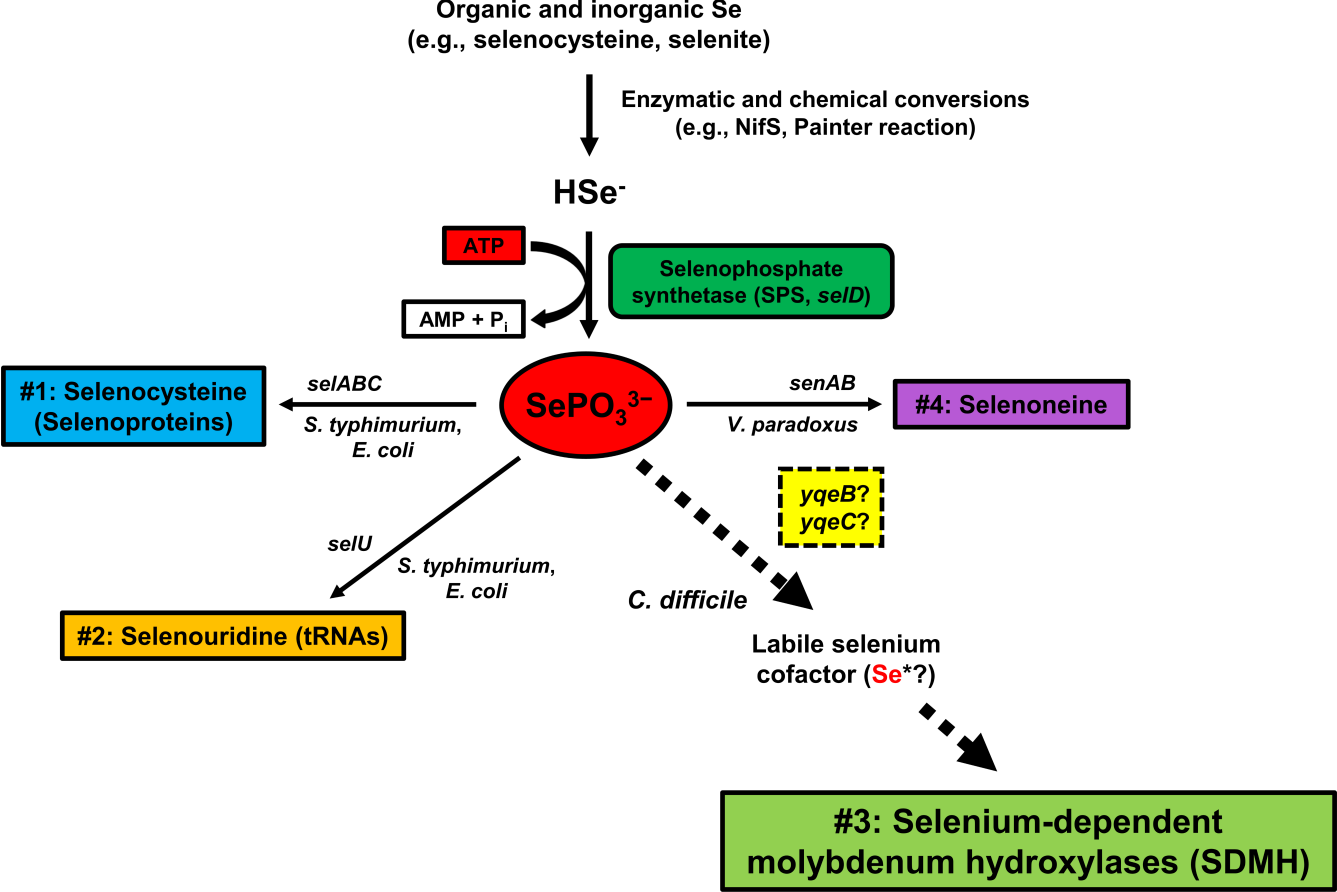
Biological pathways for specific incorporation of selenium into macromolecules. The biological insertion of selenium requires the ATP-dependent formation of selenophosphate (SePO_3_^3−^) which is an activated selenium donor necessary for the biosynthesis of selenocysteine (pathway #1) (2–5), selenouridine (pathway #2) (2–5, 9), and selenoneine (pathway #4) (11). Of all four pathways, the insertion of selenium as a labile cofactor into SDMH enzymes (pathway #3) is the least understood. While not experimentally proven, the *yqeB* and *yqeC* genes are assumed to be necessary for this selenium utilization trait (15, 16). For each pathway, the genes required for selenium insertion and the organism in which the system was characterized are indicated above and below each arrow, respectively. This figure was adapted from a figure that was originally published by the senior author of this work (17). This chapter was published in Comprehensive Natural Products, Vol. 2, Self, W.T., 121–148. Copyright Elsevier (2010).

Selenium is an important trace element generally involved in catalyzing redox reactions in all domains of life. The biological utilization of selenium is achieved by its specific incorporation into molecules with unique chemical properties (Fig. 1). The best-characterized use of selenium is the biosynthesis of the 21st amino acid selenocysteine and its subsequent incorporation into special oxidoreductases known as selenoproteins, which are generally involved in redox homeostasis and energy metabolism (1). First characterized in *Salmonella typhimurium* and *Escherichia coli* (2–5), bacterial selenoprotein synthesis is a co-translational process that requires the combined actions of the selenocysteine synthase SelA (*selA*), the selenocysteine-specific elongation factor SelB (*selB*), the selenocysteine-specific tRNA^Sec^ (*selC*), and the *cis*-acting selenocysteine insertion sequence (SECIS) element (3, 6, 7). Besides selenoproteins, selenium is also incorporated into nucleic acids and small molecules through lesser-known pathways mainly illustrated in bacteria to date. Regarding nucleic acids, the selenouridine synthase SelU (*selU*) is required to replace 2-thiouridine with 2-selenouridine in the wobble positions of bacterial and archaeal tRNAs (8–10). In terms of small molecules, the production of selenoneine was recently characterized *in vitro* using the purified enzymes selenoneine synthase SenA (*senA*) and selenosugar synthase SenB (*senB*) from *Variovorax paradoxus* (11) though this has not been demonstrated *in vivo*. While the role of 2-selenouridine and selenoneine in bacterial physiology is still unclear, the gene products required for each utilization trait clearly manipulate selenium in a biologically purposeful manner. Specific incorporation of selenium ultimately requires the formation of the activated selenium donor selenophosphate (12, 13), which is produced from the ATP-dependent phosphorylation of selenide via the selenophosphate synthetase SelD (*selD*, alternatively *senC* in *V. paradoxus*) (14). Indeed, selenophosphate plays a central role in the biological utilization of selenium as it is ultimately essential for the specific production of selenocysteine (2–5), selenouridine (2–5, 9), and selenoneine (11).

In addition to the above utilization traits, selenium is uniquely found as a labile cofactor in the active sites of some bacterial molybdoenzymes, specifically xanthine dehydrogenase (XDH) (18, 19), purine hydroxylase (PH) (19, 20), and nicotinic acid hydroxylase (21, 22). Originally purified and characterized from *Gottschalkia acidiurici* (formerly *Clostridium*) (23, 24), *Clostridium cylindrosporum* (23, 25), *Eubacterium barkeri* (formerly *Clostridium*) (18, 26, 27), and *Gottschalkia purinilytica* (formerly *Clostridium purinolyticum*) (19, 20), these proteins belong to a class of enzymes known as selenium-dependent molybdenum hydroxylases (SDMHs), which are involved in the catabolism of nicotinic acid and purines (e.g., hypoxanthine, xanthine, and uric acid) for carbon and nitrogen (26, 28, 29). Molybdenum hydroxylases, such as the bovine xanthine oxidoreductase (XOR), catalyze the hydroxylation of carbon substrates (e.g., purines and nicotinate) using water as the hydroxyl oxygen donor (30). Generally, molybdenum hydroxylases contain multiple redox centers including a molybdenum center, flavin adenine dinucleotide (FAD), and iron-sulfur (FeS) clusters (30, 31). In most XORs, the molybdenum center typically coordinates a terminal sulfur atom which is essential for catalytic activity but also extremely labile (32). Notably, cyanide inactivates the enzyme through the forced release of the sulfur (known as the “cyanolyzable sulfur”) from the active site as thiocyanate; moreover, reconstitution of enzymatic activity can be achieved by incubation of the cyanolyzed enzyme with excess sulfide (32, 33). Analogous to the cyanolyzable sulfur, the labile selenium cofactor in SDMHs is also required for catalysis (18, 19, 34) though it is not entirely clear why the clostridial enzymes utilize selenium in place of sulfur. Based on the significant difference in turnover numbers between sulfur-dependent enzymes (e.g., bovine XOR: 15 s^−1^) (35) and selenium-dependent enzymes (e.g., *G. purinilytica* PH: 412 s^−1^) (20), it is thought that selenium offers a superior catalytic advantage likely exploited by the purinolytic clostridia (22). Nevertheless, despite the wealth of biochemical information on SDMHs, there are still several considerable gaps in knowledge about the nature of the labile selenium cofactor, specifically regarding the unknown mechanism of its integration into SDMHs and whether the selenium is even derived from selenophosphate.

A genetic model for the biological utilization of selenium is necessary to elucidate the mechanism of insertion and maturation of the labile selenium cofactor in SDMHs though the purinolytic clostridia are poor candidates due to their limited genetics. Furthermore, while *E. coli* serves as a model organism for many biological processes, it likely does not make SDMHs and only possesses an incomplete purine degradation pathway (36, 37). An alternative organism must, therefore, be considered. The nosocomial pathogen *Clostridioides difficile* (formerly *Clostridium*) is the leading cause of antibiotic-associated diarrhea (38). *C. difficile* colonizes the large intestine and causes disease by the action of its exotoxins TcdA and TcdB (39). We believe that *C. difficile* is an appropriate organism to serve as a genetic model to probe this pathway because of its clear reliance on selenium (40, 41), genetic tractability (42), and similarity to the purinolytic clostridia from which SDMHs were first characterized. In this study, we take the first steps toward genetic characterization of the selenium cofactor utilization trait by providing genetic evidence for a selenium-dependent purine degradation pathway in *C. difficile*. Moreover, we contribute further knowledge to the field by investigating the role of two genetic markers for the selenium cofactor trait (*yqeB* and *yqeC*) in *C. difficile* purine catabolism.

## RESULTS

### *C. difficile* contains gene clusters that putatively encode molybdenum hydroxylases

To verify if *C. difficile* possesses the genetic potential to utilize the labile selenium cofactor, we first sought out putative SDMH genes in the *C. difficile* genome. Having previously shown that the EF2570 gene in *Enterococcus faecalis* V583 encodes a selenium-dependent XDH (43), we performed tblastn of EF2570 against the *C. difficile* 630 and R20291 genomes to search for open reading frames (ORFs) encoding potential SDMHs. From our BLAST analysis, we identified five loci each consisting of genes encoding putative subunits for multiple molybdenum hydroxylases (Fig. S1). Genes in these loci have been previously reported by our group and others via different methods of computational biology (15, 16, 44, 45). Molybdenum hydroxylases are generally considered to harbor subunits with a molybdenum cofactor, FAD, and FeS clusters (30, 31). In *E. coli*, the genes encoding these subunits are typically annotated as *xdhA*, *xdhB*, and *xdhC*, respectively (36). Since the *C. difficile* gene products have not yet been characterized, we tentatively used this *E. coli* nomenclature for convenience. All five loci contained genes predicted to encode for molybdenum cofactor-binding subunits (annotated as *xdhA1* through *xdhA5*), but not all of them contained genes encoding FAD-binding and FeS-containing subunits. In fact, only three genes encoding hypothetical FAD-binding subunits (annotated as *xdhB1* through *xdhB3*) were found to colocalize with *xdhA1*, *xdhA2*, and *xdhA4*. Similarly, three genes encoding hypothetical FeS-containing subunits (annotated as *xdhC1* through *xdhC3*) were found in our analysis, and they colocalized with *xdhA2* (and *xdhB*2), *xdhA3*, and *xdhA4* (and *xdhB3*). The gene IDs and genomic locations of these ORFs are listed in Table S1. Four out of five loci (*xdhA1* through *xdhA4*) were found within close proximity to each other while the fifth locus (*xdhA5*) was isolated elsewhere on the chromosome. Additionally, *xdhA5* is uniquely predicted to simultaneously encode both a molybdenum center and an FeS-containing subunit based on sequence identity to EF2570. The diversity of these gene clusters suggests that some molybdenum hydroxylases may exhibit unique substrate specificities depending on the presence or absence of certain redox centers (e.g., FAD and FeS). Overall, the presence of these molybdenum hydroxylase gene clusters caused us to speculate that *C. difficile* could utilize selenium to modify these putative molybdoenzymes into SDMHs.

### Hypoxanthine, xanthine, and uric acid enhance *C. difficile* growth in a minimal medium lacking glycine and threonine

**Fig 2:**
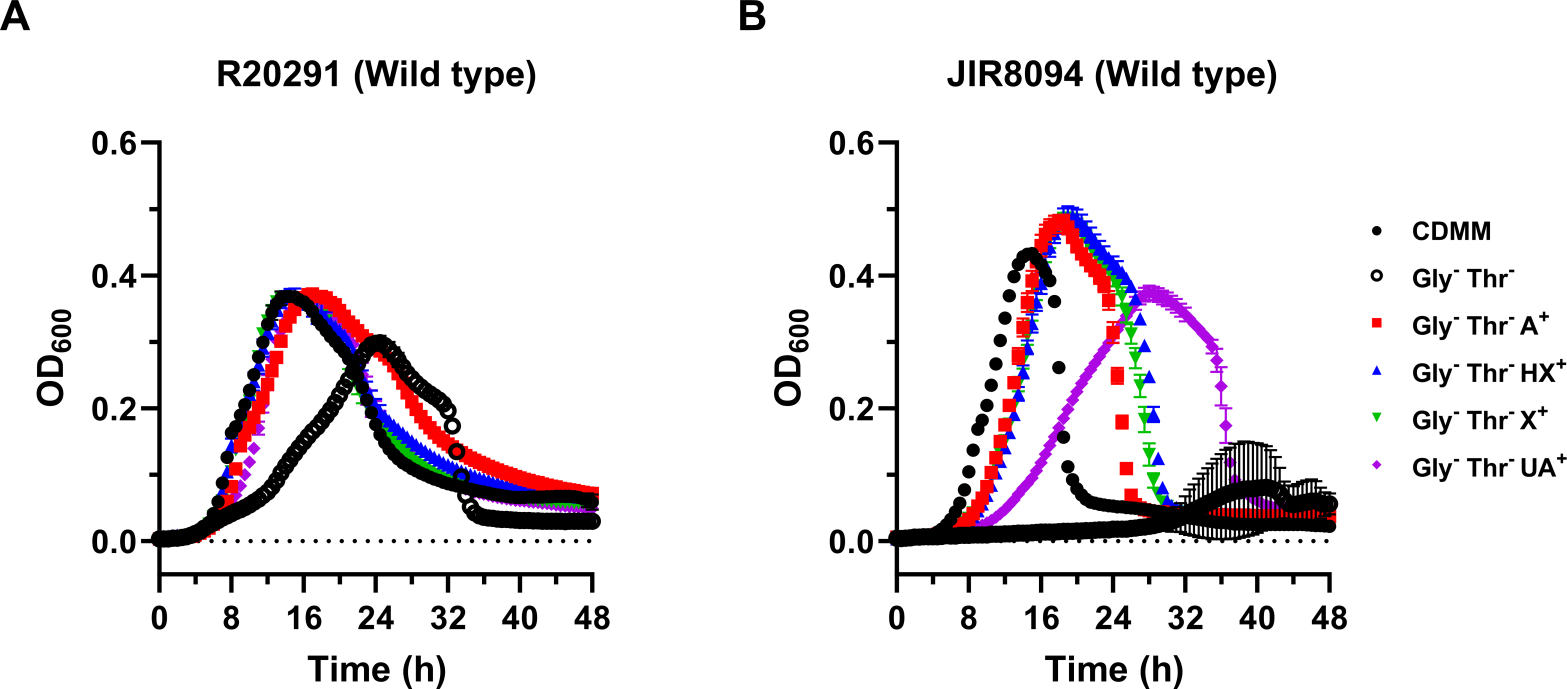
Hypoxanthine, xanthine, and uric acid induce rapid growth of *C. difficile* in a minimal medium devoid of glycine and threonine. *C. difficile* wild-type strains (**A**) R20291 and (**B**) JIR8094 were grown in CDMM at 37°C for 48 h. The turbidity (OD_600_) of each culture was recorded every 0.5 h over the 48 h period. When indicated, glycine and threonine were omitted (Gly^−^ Thr^−^) and substituted with 1 mM adenine (A^+^), hypoxanthine (HX^+^), xanthine (X^+^), or uric acid (UA^+^). The experiment was repeated twice. Data points represent the means of triplicate cultures, while error bars represent standard deviations.

Based on the fact that various soil clostridia catalyze purinolytic reactions using SDMHs (29), we wondered if *C. difficile* could likewise catabolize known SDMH substrates such as hypoxanthine, xanthine, and uric acid. However, when grown in rich (BHIS) or minimal (CDMM) media separately augmented with each purine at 1 mM, *C. difficile* strains R20291 and JIR8094 exhibited no significant differences in growth pattern (Fig. S2). While this initially suggested that *C. difficile* does not benefit from the addition of these purines, we reasoned that significant purine-dependent changes in physiology could not be observed under these growth conditions. In the seminal report that describes the original recipe for CDMM, Karasawa et al. (46) observed that *C. difficile* VPI 10463—which was found to grow poorly in the absence of glycine and threonine—could use adenine to compensate for both amino acids. This finding inspired us to design growth experiments centered on this unique physiological phenomenon in order to study the effects of purine catabolism, so we first evaluated whether this adenine-dependent effect occurred in our strains as well. Indeed, we successfully recapitulated the finding with R20291 and JIR8094: both suffered similar growth defects in the absence of glycine and threonine while substitution of both amino acids with adenine sufficiently resulted in growth levels identical to the CDMM controls (Fig. 2). Using this unique growth behavior as a proxy for growth on purines, we repeated the assay with hypoxanthine, xanthine, and uric acid and, indeed, observed similar enhanced growth with each purine in the absence of glycine and threonine (Fig. 2), suggesting that they were also compensating for the deficiency of these amino acids. Interestingly, while R20291 responded identically to every tested purine (Fig. 2A), JIR8094 did not grow as well on uric acid as compared to the other purines (Fig. 2B). Nevertheless, these results suggest that *C. difficile* utilizes hypoxanthine, xanthine, and uric acid as growth substrates.

### Selenophosphate synthetase plays a major role in growth with xanthine and uric acid but not hypoxanthine

**Fig 3:**
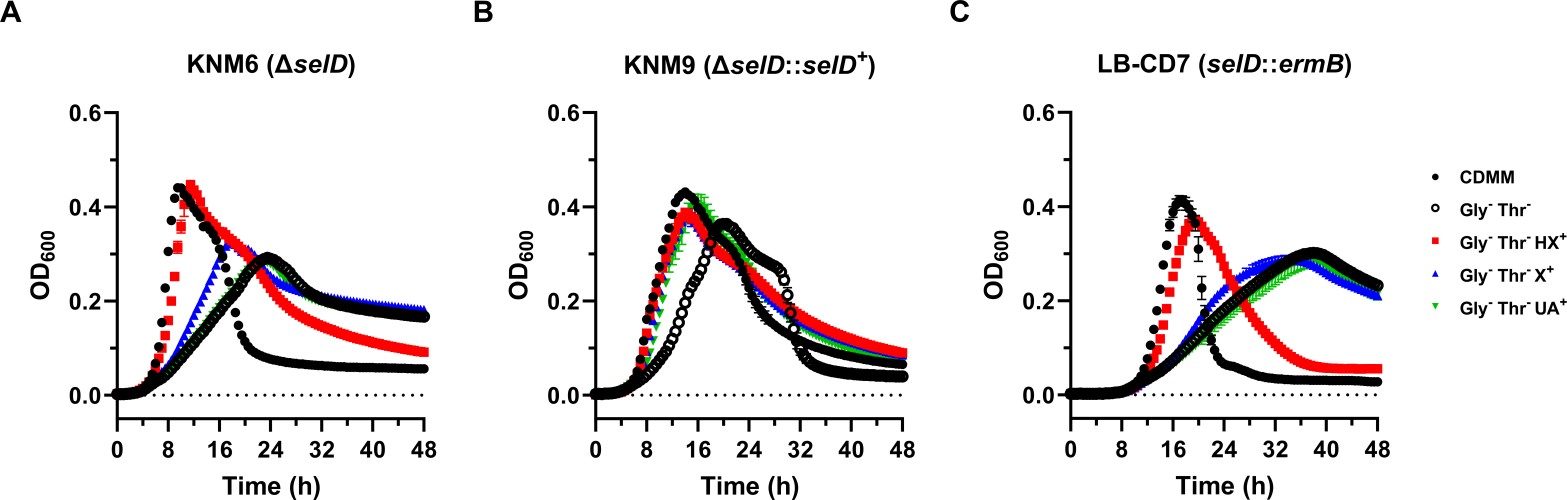
Selenophosphate synthetase is required for rapid growth with xanthine and urate but not hypoxanthine in the absence of glycine and threonine. *C. difficile selD* mutant strains (**A**) KNM6, (**B**) KNM9, and (**C**) LB-CD7 were grown in CDMM at 37°C for 48 h. The turbidity (OD_600_) of each culture was recorded every 0.5 h over the 48 h period. When indicated, glycine and threonine were omitted (Gly^−^ Thr^−^) and substituted with either 1 mM hypoxanthine (HX*^+^*), xanthine (X*^+^*), or uric acid (UA*^+^*). The experiment was repeated twice. Data points represent the means of triplicate cultures, while error bars represent standard deviations.

To determine if these purine-dependent growth patterns relied on the production of selenophosphate, we repeated the assay with *selD* mutant strains KNM6 (Δ*selD*; R20291 background) and LB-CD7 (*selD::ermB*; JIR8094 background). We found that both *selD* mutants were unable to utilize uric acid for rapid growth compared to wild-type strains independent of genetic background (Fig. 3A and C). Moreover, growth on xanthine was severely impaired but not completely abolished (Fig. 3A and C). The restored *selD* mutant strain KNM9 (Δ*selD::selD*^+^; KNM6 background) did not suffer a growth defect in the presence of xanthine and uric acid, instead exhibiting a behavior similar to the wild-type R20291 (Fig. 3B). In contrast, mutation of *selD* did not affect growth on hypoxanthine as all mutants grew as well as wild-type strains (Fig. 3). These results suggest that selenophosphate synthetase is absolutely required for uric acid utilization, partially required for xanthine utilization, and not required for hypoxanthine utilization.

### *yqeB* and *yqeC* are putative genetic determinants for the maturation of the labile Se cofactor

**Fig 4:**
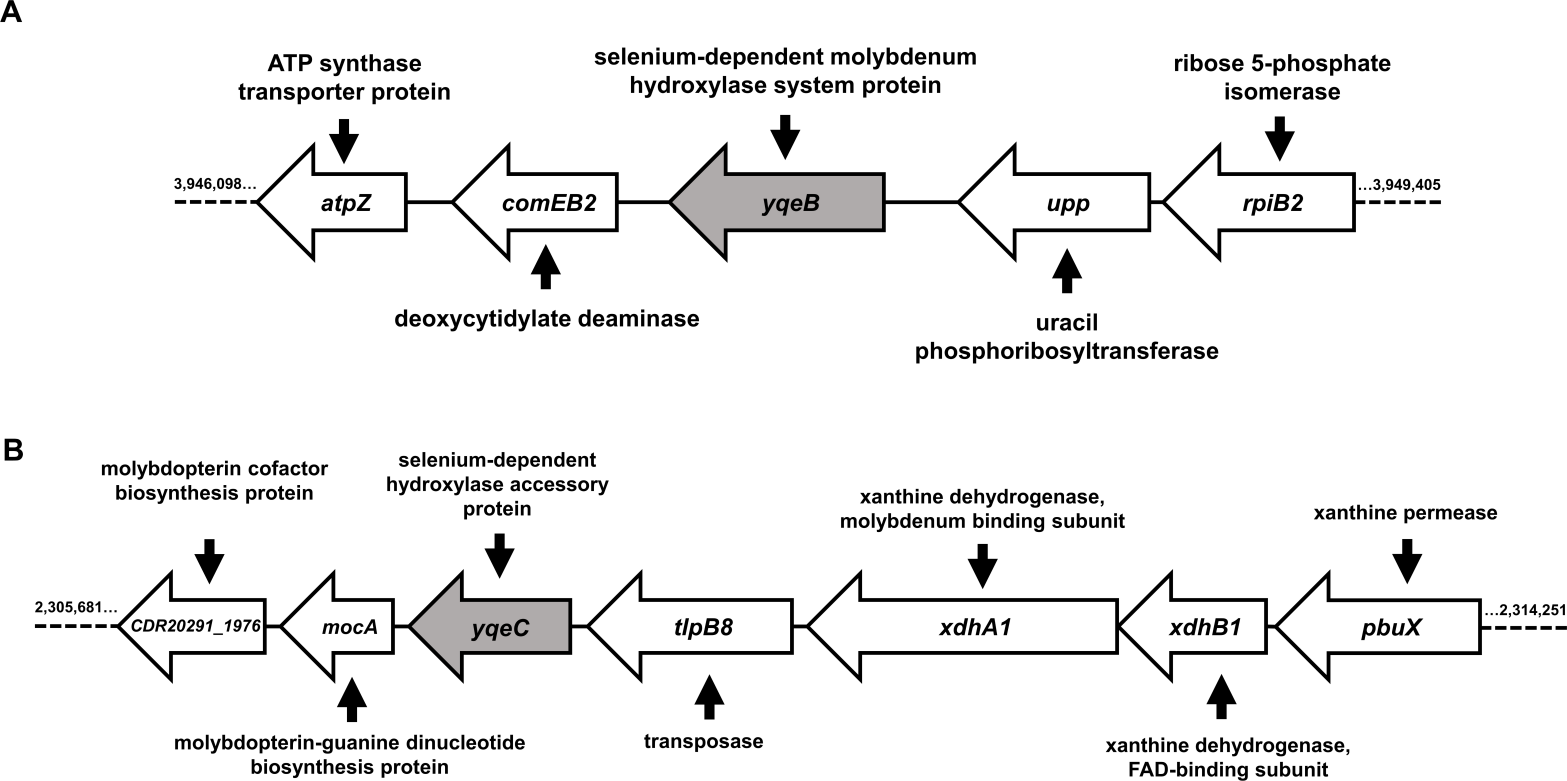
*yqeB* and *yqeC* are located within gene clusters associated with pyrimidine and purine metabolism in *C. difficile*. The markers for the SDMH trait (*yqeB* and *yqeC*) are present within the *C. difficile* genome, but they do not co-localize with each other or with *selD* as in other bacterial genomes (15, 16). (**A**) *yqeB* is flanked by two genes putatively involved in pyrimidine metabolism. (**B**) *yqeC* is located among genes associated with the biosynthesis of a molybdenum hydroxylase. Numbers on each side of the gene clusters indicate the location within the R20291 genome.

Given that SelD plays a role in enhancing the growth on purines, we speculated that SDMHs may be involved in this process. While the mechanism by which the labile Se cofactor is inserted into these molybdoenzymes is unknown, our lab and another group previously identified two uncharacterized genes—*yqeB* and *yqeC*—that appear to act as markers for the SDMH trait based on their co-localization with *selD* and gene clusters encoding molybdenum hydroxylases in several bacterial species including *E. faecalis* (15, 16). Both genes were also identified in the *C. difficile* genome (15, 16) though they did not co-localize with each other or with *selD* (Fig. 4). Instead, *yqeB* (CD630_34780; CDR20291_3314) was flanked by two genes predicted to encode enzymes apparently involved in pyrimidine metabolism (*upp*, uracil phosphoribosyltransferase; *comEB2*, deoxycytidylate deaminase) (Fig. 4A). On the other hand, *yqeC* (CD630_20710; CDR20291_1978) was located closely downstream of the *xdhA1-xdhB1* gene cluster and upstream of genes encoding proteins putatively involved in molybdenum cofactor biosynthesis (*mocA*, molybdopterin-guanine dinucleotide biosynthesis protein; CDR20291_1976, molybdopterin cofactor biosynthesis protein) (Fig. 4B). Because of their reported association with the SDMH utilization trait (15, 16), we wondered if these genes played any role in *C. difficile* purine catabolism.

### *yqeB* plays a substantial role in growth on xanthine and uric acid but not hypoxanthine

**Fig 5:**
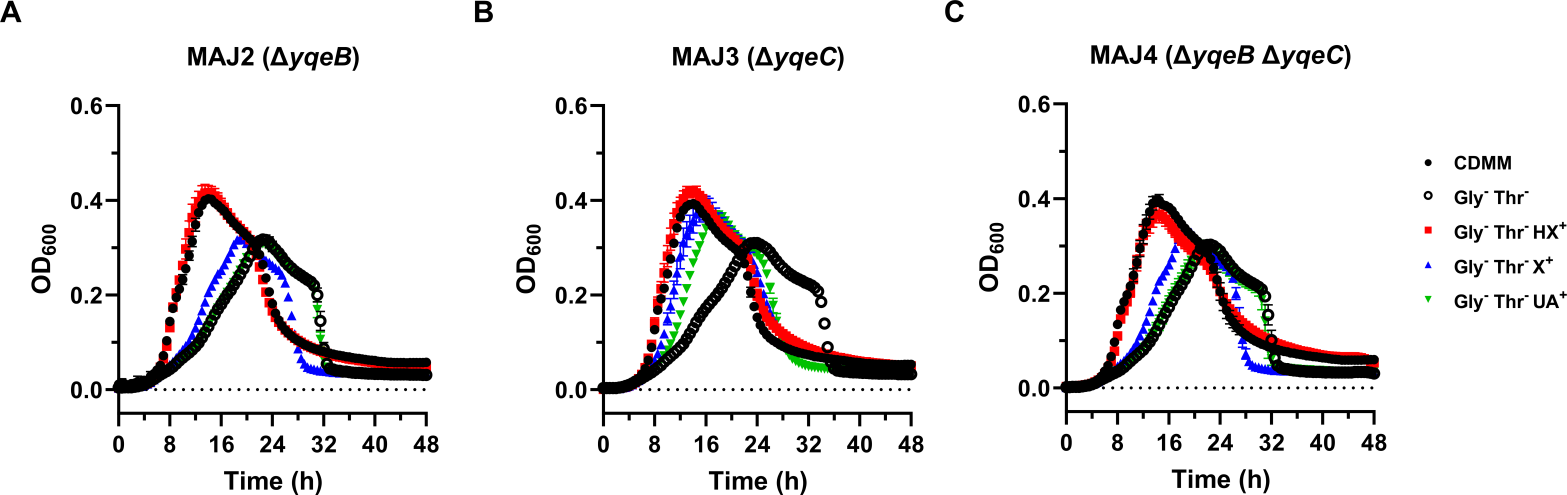
The product of *yqeB* but not *yqeC* is necessary for rapid growth with uric acid or xanthine in the absence of glycine and threonine. *C. difficile yqeB* and *yqeC* mutant strains (**A**) MAJ2, (**B**) MAJ3, and (**C**) MAJ4 were grown in CDMM at 37°C for 48 h. The turbidity (OD_600_) of each culture was recorded every 0.5 h over the 48 h period. When indicated, glycine and threonine were omitted (Gly^−^ Thr^−^) and substituted with 1 mM hypoxanthine (HX^+^), xanthine (X^+^), or uric acid (UA^+^). The experiment was repeated twice. Data points represent the means of triplicate cultures, while error bars represent standard deviations.

To determine the role of these two genes in purine catabolism, we used a recently published dual-plasmid CRISPR-Cas9 system to delete *yqeB* and *yqeC* from the R20291 genome (47). After conjugation of the Cas9-encoding plasmid pJB06 into R20291, this strain was then used as the recipient for subsequent conjugations of newly constructed targeting plasmids for *yqeB* (pMJ18) and *yqeC* (pMJ21). Xylose induction of CRISPR-Cas9 machinery resulted in the generation of mutant strains MAJ2 (Δ*yqeB*), MAJ3 (Δ*yqeC*), and MAJ4 (Δ*yqeB* Δ*yqeC*) as verified by colony PCR with primers flanking the mutation sites in the chromosome (Fig. S3). Growth of these mutants in BHIS and CDMM revealed no obvious growth phenotypes (Fig. S4). However, in CDMM lacking glycine and threonine, all mutants suffered the same growth defect as R20291 (Fig. 5). The addition of uric acid was unable to enhance the growth of the Δ*yqeB* mutant (Fig. 5A), suggesting that it could no longer utilize the purine as a growth substrate. Moreover, growth of the Δ*yqeB* mutant in the presence of xanthine was severely diminished though it could still fully benefit from hypoxanthine (Fig. 5A). These Δ*yqeB* growth phenotypes were very similar to the growth phenotypes exhibited by the *selD* mutants (Fig. 3A and C), indicating that both genes are equally necessary for this process. Indeed, the xanthine and uric acid phenotypes of the Δ*yqeB* mutant were fully complemented by a plasmid containing a wild-type copy of *yqeB* under the control of its native promoter (pMJ23) compared to the empty vector control (pHN149) (Fig. S5A and B). In contrast, the Δ*yqeC* mutant showed no appreciable growth change in all tested conditions (Fig. 5B), implying that the *yqeC* gene product does not play a necessary role in these growth conditions. In further support of this idea, the double mutant Δ*yqeB* Δ*yqeC* was identical to the Δ*yqeB* mutant in that it did not benefit from uric acid, barely grew better with xanthine, and fully exploited hypoxanthine (Fig. 5C). However, we surprisingly observed only partial complementation of the xanthine and uric acid phenotypes in the Δ*yqeB* Δ*yqeC* mutant containing pMJ23 compared to empty vector control (Fig. S5C and D), hinting that *yqeC* is still required for optimal utilization of these purines. Overall, these results heavily suggest that *yqeB* plays a necessary role in selenium-dependent purine catabolism, while the role of *yqeC* remains uncertain.

## DISCUSSION

The biological utilization of selenium is a well-established genetic system present in all domains of life. Although many pathways of selenium incorporation have been genetically characterized such as selenocysteine biosynthesis, the biological mechanism by which molybdenum hydroxylases are modified with selenium is poorly understood (Fig. 1). Despite decades of biochemical and spectroscopic research on SDMHs, the field has remained stagnant due to the fact that the selenium cofactor trait lacks genetic characterization. In this work, we lay the foundation for further characterization of this trait by providing genetic evidence of selenium-dependent purine degradation in *C. difficile*. Specifically, we observed that two *selD* mutants from different strain lineages exhibited impaired growth on xanthine and uric acid in the absence of glycine and threonine (Fig. 3A and C). If *C. difficile* does indeed use SDMHs to degrade these purines, our results strongly suggest that selenophosphate synthetase is required for this process. As it is still unknown whether the labile selenium cofactor even originates from selenophosphate, our observation of SelD-dependent growth on two known SDMH substrates may help to answer this fundamental question.

In this study, we examined the role of *yqeB* and *yqeC* in purine-dependent growth of *C. difficile*. We found that *yqeB* was required for optimal growth on xanthine and uric acid in the absence of glycine and threonine (Fig. 5A), suggesting that the *yqeB* gene product is just as important as selenophosphate synthetase for this process. Comparatively, deletion of *yqeC* gave no apparent phenotype (Fig. 5B) while the Δ*yqeB* Δ*yqeC* double mutant exhibited a phenotype that was no different from the Δ*yqeB* mutant (Fig. 5C), implying that the *yqeC* gene product is dispensable for this catabolic system. Given the fact that the mere co-existence of *yqeB* and *yqeC* appears to function as a genetic marker for the selenium cofactor trait (15, 16), we found this apparent inequality between the two genes puzzling. Several studies, however, appear to hint at a physiological “preference” of *yqeB* over *yqeC*. For example, in multidrug-resistant *E. faecalis* MMH594, EF2563 (*yqeB*) was one of many selenium- and molybdenum-associated genes essential for growth in Mueller-Hinton broth according to transposon insertion sequencing (48). *E. faecalis* is a selenium-utilizing organism that exclusively harbors the selenium cofactor trait based on the presence and co-localization of *yqeB*, *yqeC*, and *selD* (15, 16), so it is intriguing that only *yqeB* was deemed essential in that study (48). In addition, several groups have identified a SigL-dependent (σ^54^) promoter upstream of *yqeB* but not *yqeC* in *C. difficile* (49, 50). This SigL-dependent promoter is predicted to be recognized by an uncharacterized bacterial enhancer-binding protein known as DioR (49). Interestingly, the proposed regulon of DioR includes *pbuX* (xanthine-specific purine permease) and *pyrC* (dihydroorotase) (49), suggesting a regulatory role in *C. difficile* purine and pyrimidine metabolism. While these observations seem to emphasize the cell’s preference of *yqeB* over *yqeC* in physiology, it must be noted that the Δ*yqeB* Δ*yqeC* double mutant was only partially complemented by a wild-type copy of *yqeB* (Fig. S5D), suggesting that *yqeC* may still be required for full utilization of xanthine and uric acid. Further biochemical studies are needed to identify the function of each gene product and clarify these observations. Unfortunately, because YqeB and YqeC are hypothetical proteins with no known predicted domains, it is difficult to envision a clear mechanism of how they contribute to the maturation of the selenium cofactor. However, we tentatively observe that *C. difficile* YqeB is 29.3% identical to an acetyl-CoA carboxylase biotin carboxyl carrier protein encoded by a gene annotated as *accB* in the genome. In *E. coli*, AccB binds biotin and presents it as a substrate for carboxylation by the acetyl-CoA carboxylase (51). If the identical region in YqeB also functions as a binding domain, it may be that this protein aids in SDMH modification by binding some to-be-determined substrate (e.g., the molybdenum cofactor or a selenium intermediate). Further biochemical studies centered on this hypothesis may help elucidate the mechanism of selenium cofactor integration.

During our investigation, we observed impaired growth of *selD* and *yqeB* mutants on xanthine and uric acid but not on hypoxanthine (Fig. 3 and 5), implying the presence of selenium-dependent and selenium-independent catabolic enzymes with varying substrate specificities. While hypoxanthine is a preferred substrate of selenium-dependent PH in *G. purinilytica* (52), *C. difficile* may instead oxidize hypoxanthine using a sulfur-dependent molybdenum hydroxylase or a completely different pathway. Additionally, we observed that *C. difficile selD* and *yqeB* mutants completely lost the growth-enhancing effect from uric acid but still partially benefited from xanthine (Fig. 3 and 5). We speculate that uric acid decomposition may require initial catalysis by an SDMH, while xanthine may function as a substrate for both sulfur-dependent and selenium-dependent enzymes. It must also be noted that the clostridial XDHs are reversible SDMHs that can not only oxidize xanthine to uric acid but also reduce urate back to xanthine (18, 25, 53). Therefore, if *C. difficile* encodes an SDMH with XDH activity that targets both substrates, an alternate hypothesis would be that urate reduction is strictly a selenium-dependent reaction, while xanthine oxidation can still proceed independently of selenium albeit at a lower rate of activity. Overall, we believe these purine-dependent growth phenotypes may arise from varying substrate specificities exhibited by different molybdenum hydroxylases. The diversity of FAD-binding and FeS-containing subunits in each molybdenum hydroxylase gene cluster may produce enzymes each with unique redox chemistry (Fig. S1), which certainly supports this hypothesis. Biochemical characterization of each putative molybdenum hydroxylase will aid in mapping out the metabolic pathways of purine catabolism in *C. difficile*.

While purine catabolism has been thoroughly studied across various clostridia and enterobacteria (28, 54), the role of purines as a nutrient source for *C. difficile* during infection has not been defined. Purines are likely to be relevant nutrients for *C. difficile* during gut colonization according to several observations. First, it is well known that the gut functions as a reservoir for uric acid considering that approximately one-third of the uric acid produced in the body is eliminated via the gastrointestinal tract (55). Second, a recent study demonstrated that hypoxanthine and xanthine are gut microbiota-derived products that are present in the intestinal lumen (56). Lastly, Girinathan et al. (57) found that various cecal nutrients including hypoxanthine were significantly enriched in gnotobiotic mice co-colonized with *C. difficile* and either *Clostridium sardiniense* or *Paraclostridium bifermentans*. In that same study, genes for xanthine metabolism and transport were found to be upregulated in *C. difficile* (57). These observations strongly reinforce the idea of host-derived purines as available nutrients for *C. difficile* during infection. Interestingly, for the purinolytic clostridia, purine degradation seems to begin at the point of xanthine, resulting in the eventual breakdown of ammonia, acetate, carbon dioxide, and formate (29). Supposing a similar biochemical scheme in *C. difficile*, xanthine could potentially be generated from hypoxanthine oxidation and urate reduction via molybdenum hydroxylases though further experiments are needed to thoroughly characterize this pathway. In support of our findings describing enhanced growth on uric acid (Fig. 2), evidence of this purine’s decomposition by *C. difficile* was recently reported by two groups attempting to identify and characterize the human gut commensals responsible for anaerobic uric acid degradation (58, 59). In the study by Kasahara et al. (58), a panel of purine-degrading bacteria enriched from human feces contained a *C. difficile* isolate (CD196) that grew on agar overlaid with saturating uric acid. Likewise, in a different panel of human gut bacteria studied by Liu et al. (59), three *C. difficile* strains (ATCC BAA-1801, M68, and 630) were able to sufficiently deplete uric acid from a carbohydrate-limited chopped meat medium; curiously, xanthine accumulated in the culture supernatants during uric acid consumption. While it is clear from these studies that *C. difficile* catabolizes uric acid, it is still unclear whether SDMHs participate in this process. If *in vivo* decomposition of uric acid proceeds via a selenium-dependent manner as inferred by our *in vitro* data (Fig. 3), it would serve as compelling evidence for the selenium cofactor trait serving a role in *C. difficile* infection. In summary, our findings provide a basis for further study of purine catabolism in *C. difficile* and may help delineate new selenium-dependent and selenium-independent mechanisms for scavenging purines.

## MATERIALS AND METHODS

### Bacterial strains, culture media, and growth conditions

Bacterial strains are listed in Table 1. *C. difficile* strains were routinely cultured in an anaerobic atmosphere (~1.0% H_2_, 5% CO_2_, ~94% N_2_) generated by a Coy anaerobic chamber. Hydrogen levels were maintained within a range of ±0.2% based on continuous detection by a Coy anaerobic monitor (CAM-12). *C. difficile* strains were grown in 37 g/L brain heart infusion (BHI) supplemented (BHIS) with 5 g/L yeast extract and 0.1% L-cysteine (60). When necessary, the following antibiotics were supplemented to BHIS: thiamphenicol (10 µg/mL), lincomycin (20 µg/mL), kanamycin (50 µg/mL), or D-cycloserine (250 µg/mL). *E. coli* strains were cultured in lysogeny broth (LB) containing 10 g/L tryptone, 5 g/L yeast extract, and 5 g/L sodium chloride (61). When necessary, the following antibiotics were supplemented to LB: ampicillin (100 µg/mL), chloramphenicol (25 µg/mL), or erythromycin (200 µg/mL). For physiological studies, *C. difficile* strains were grown in CDMM (46), which was prepared in a manner described previously (62). When indicated, glycine and threonine were omitted from CDMM preparations and substituted with adenine, hypoxanthine, xanthine, or uric acid at 1 mM from autoclaved 20 mM stock solutions. To make these solutions, solid purines were suspended in hot deionized water (containing phenol red to monitor pH) and eventually dissolved by slow addition of sodium hydroxide from a 1 M solution. For *yqeB* complementation tests, thiamphenicol was included in all growth media to maintain complementation plasmids.

**TABLE 1:** Bacterial strains used in this study

Bacterial strain	Relevant genotype or description	Reference/source
*E. coli*		
NEB 5-alpha	*fhuA2* Δ(*argF-lacZ)U169 phoA glnV44* Φ*80*Δ(*lacZ)M15 gyrA96 recA1 relA1 endA1 thi-1 hsdR17*	New England Biolabs
HB101 pRK24	*lacYI galK2 xyl-6 mtl-I repsL20* pRK24 (Amp^R^)	(40)
*C. difficile*		
R20291	Wild type, ribotype 027	(40)
KNM6	R20291 (Δ*selD*) CRISPR-Cas9 mutant	(40)
KNM9	KNM6 (Δ*selD*::*selD*^+^) CRISPR-Cas9 mutant	(41)
MAJ2	R20291 (Δ*yqeB*) CRISPR-Cas9 mutant	This study
MAJ3	R20291 (Δ*yqeC*) CRISPR-Cas9 mutant	This study
MAJ4	MAJ2 (Δ*yqeB* Δ*yqeC*) CRISPR-Cas9 mutant	This study
JIR8094	Wild type, ribotype 012, Erm^S^ derivative of strain 630	(63)
LB-CD7	JIR8094 (*selD*::*ermB*) TargeTron mutant	(40)

### Growth studies and analysis

Growth studies were performed as done previously with slight modifications (62). Briefly, single colonies of *C. difficile* strains were inoculated into 5 mL BHIS broths and grown at 37°C for 16–24 h. Overnight BHIS cultures were diluted 100-fold the following day in 5 mL CDMM broths, which were then grown at 37°C for 16–24 h. Overnight CDMM cultures were diluted 100-fold the following day in test media which were then transferred to sterile 96-well plates in 200 µL triplicate volumes. Diluted cultures were incubated at 37°C for 48 h in a BioTek Epoch 2 Microplate Spectrophotometer. Growth was monitored by turbidity measurements (OD_600_) recorded every 0.5 h over the 48 h period. Before each OD_600_ measurement, cultures were rapidly resuspended for 5 s using the double orbital function on the fast setting.

### Plasmid construction

All plasmids are listed in Table 2. Primers are listed in Table S2. The targeting plasmids for *yqeB* and *yqeC* were constructed using pJB07 as a template (47). In order to generate clean in-frame deletions of *yqeB* and *yqeC* in the R20291 chromosome (i.e., deletion of entire ORF including start and stop codons), the pre-existing *pyrE* homology arms in pJB07 were first deleted via ‘Round-the-horn site-directed mutagenesis (64) using primers pJB07 empty FWD and pJB07 empty REV, ultimately producing a linear vector lacking homology arms. The linear vector was treated with DpnI to eliminate the remaining plasmid template. The *yqeB* homology arms were generated by PCR amplification of R20291 genomic DNA with primers flanking the 500 bp regions directly upstream (yqeB-UA-fwd and yqeB-UA-rev) and downstream (yqeB-DA-fwd and yqeB-DA-rev) of *yqeB*. Likewise, the *yqeC* homology arms were PCR-amplified from the R20291 genome with primers flanking the 500 bp upstream regions (yqeC-UA-fwd and yqeC-UA-rev) and downstream regions (yqeC-DA-fwd and yqeC-DA-rev) of *yqeC*. In separate three-fragment assembly reactions for *yqeB* and *yqeC*, the upstream and downstream homology arms were fused together and inserted into the linear targeting vector using NEBuilder HiFi DNA Assembly (New England Biolabs) according to the manufacturer’s instructions. Assembly reactions were subsequently transformed into *E. coli* NEB 5-alpha to generate pMJ15 (for *yqeB*) and pMJ19 (for *yqeC*). In order to direct Cas9 to target *yqeB* and *yqeC*, we utilized ‘Round-the-horn site-directed mutagenesis to mutate the pre-existing *pyrE* guide RNA (gRNA) sequence in pMJ15 and pMJ19 to new gRNA sequences corresponding to *yqeB* and *yqeC*, respectively. Specifically, pMJ15 was mutated with primers yqeB gRNA 3 RTH and pJB07 gRNA rev RTH, while pMJ19 was mutated with primers yqeC gRNA 2 RTH and pJB07 gRNA rev RTH. Prior to these PCRs, ‘Round-the-horn primers used for gRNA mutation were phosphorylated with T4 polynucleotide kinase (New England Biolabs) to allow for eventual ligation of PCR products with T4 DNA ligase (New England Biolabs) as done previously (65). Linear ‘Round-the-horn PCR products were treated with DpnI to digest the remaining plasmid template, ligated overnight at room temperature using T4 DNA ligase, and subsequently transformed into NEB 5-alpha to yield pMJ18 (for *yqeB*) and pMJ21 (for *yqeC*). Plasmid constructs were confirmed via Sanger sequencing (GENEWIZ).

**TABLE 2:** Plasmids used in this study

Plasmid	Relevant genotype or features	Reference/source
pJB06	Cas9-encoding plasmid, *catP*	(47)
pJB07	Homology- and gRNA-encoding plasmid (*pyrE* homology arms, *pyrE* gRNA), *ermB*	(47)
pMJ15	pJB07 (*yqeB* homology arms, *pyrE* gRNA)	This study
pMJ18	pMJ15 (*yqeB* homology arms, *yqeB* gRNA)	This study
pMJ19	pJB07 (*yqeC* homology arms, *pyrE* gRNA)	This study
pMJ21	pMJ19 (*yqeC* homology arms, *yqeC* gRNA)	This study
pHN149	Shuttle vector used for complementation, *catP*	(66)
pMJ23	pHN149 (*yqeB* complementation plasmid)	This study

To generate the *yqeB* complementation plasmid, the *yqeB* gene under the control of its native promoter was subcloned into pHN149 (66). Briefly, the multiple cloning site of pHN149 was deleted with ‘Round-the-horn site-directed mutagenesis using primers pHN149 empty fwd and pJB07 empty REV in order to generate an empty vector. The empty ‘Round-the-horn product was treated with DpnI. PCR amplification of the *yqeB* gene (plus 300 bp directly upstream and 100 bp directly downstream) from the R20291 genome was achieved using primers yqeB comp fwd and yqeB comp rev. The *yqeB* fragment was subcloned into the empty vector using NEBuilder HiFi DNA assembly as described above. The assembly reaction was transformed into NEB 5-alpha to generate the *yqeB* complementation plasmid pMJ23.

### Conjugation into *C. difficile*

Plasmids intended for conjugation were first transformed into *E. coli* HB101 pRK24 to be used as donors. In all conjugations, *E. coli* HB101 pRK24 harboring each plasmid was grown overnight at 37°C in LB with ampicillin and either chloramphenicol for pJB06 or erythromycin for pMJ18 and pMJ21. For conjugation of pJB06, *C. difficile* R20291 was the recipient and was grown overnight at 37°C in BHIS. For conjugation of *yqeB* and *yqeC* targeting plasmids, *C. difficile* R20291 pJB06 was the recipient and was similarly grown overnight at 37°C in BHIS with thiamphenicol. Conjugation of CRISPR-Cas9 plasmids into *C. difficile* was performed using a filter-mating technique as described previously (67). Briefly, *E. coli* donor cells from overnight cultures were harvested (0.5 mL) at 5,000 × *g* for 1 min and washed with 1 mL LB to remove antibiotics. Washed donor pellets were transferred into the anaerobic chamber. *C. difficile* recipient cells were aliquoted (200 µL) into 1 mL microcentrifuge tubes, harvested at 5,000 × *g* for 1 min, and washed with an equal volume of BHIS if necessary to remove antibiotics. This harvest-and-wash step was skipped entirely if *C. difficile* was grown without antibiotics. *C. difficile* culture aliquots were incubated in a water bath at 48°C for 5 min to heat-shock the cells (67, 68). Afterward, heat-shocked *C. difficile* cultures were transferred back to the anaerobic chamber and were used to resuspend (100 µL) the donor pellets. Mixed cultures were then plated (100 µL) directly onto sterile 0.45 µm mixed cellulose filters (Millipore; HAWP02500) aseptically placed onto BHI agar as described previously (67). Once the 100 µL spots dried, the inoculated BHI plates were incubated at 37°C overnight. The following day, the resulting growth was resuspended with 0.5 mL BHIS, plated directly onto BHIS agar (with selection for the *C. difficile* transconjugants and counterselection for the *E. coli* donor), and subsequently incubated at 37°C for 24–72 h. For conjugation of pJB06 into R20291, the growth resuspension was plated onto BHIS agar with thiamphenicol, kanamycin, and D-cycloserine. For conjugation of pMJ18 and pMJ21 into R20291 pJB06, the growth resuspension was plated onto BHIS agar with the same antibiotics plus lincomycin. In both cases, transconjugants were re-streaked onto identical antibiotic-supplemented BHIS and were subsequently tested for the presence of pJB06 and each targeting plasmid via colony PCR. pJB06 was confirmed via amplification of plasmid-born *catP* using primers catP pJB06 FWD and catP pJB06 REV. pMJ18 and pMJ21 were confirmed via amplification of plasmid-born *ermB* using primers ermB pJB07 FWD and ermB pJB07 REV. Colonies were confirmed to be *C. difficile* via amplification of genomic *tcdB* using primers tcdB FWD and tcdB internal rev. Confirmed transconjugants were used for downstream experimentation. For conjugation of complementation plasmids pHN149 and pMJ23, the above procedure was performed as previously stated, but the recipients were *C. difficile* MAJ2 and MAJ4. pHN149 and pMJ23 were maintained with chloramphenicol in *E. coli* and thiamphenicol in *C. difficile*. Conjugation of each plasmid was confirmed via amplification of plasmid-born *catP* as above using primers catP pJB06 FWD and catP pJB06 REV.

### CRISPR induction

To induce Cas9-mediated deletion of *yqeB* and *yqeC*, single colonies of *C. difficile* R20291 pJB06 containing either pMJ18 or pMJ21 were streaked onto BHIS supplemented with thiamphenicol, lincomycin, and 1% xylose. Plates were incubated at 37°C until isolated colonies arose (typically ~24–72 h). Colonies were continually passaged onto identical media up to five times until normal growth rates were restored. At this stage, colonies were screened for successful mutation using colony PCR. Deletion of *yqeB* was confirmed using primers yqeB mutation check fwd and yqeB mutation check rev. Deletion of *yqeC* was confirmed using primers yqeC mutation check fwd and yqeC mutation check rev. Confirmed mutant colonies were cured of CRISPR-Cas9 plasmids through inoculation of BHIS broth supplemented with 2% xylose and subsequent overnight growth at 37°C. A loopful of overnight culture was streaked for isolation on non-selective BHIS and grown overnight at 37°C, and resulting colonies were pick-and-patched onto BHIS with and without thiamphenicol and lincomycin. Thiamphenicol- and lincomycin-sensitive isolates were confirmed for plasmid loss via colony PCR using primers catP pJB06 FWD, catP pJB06 REV, ermB pJB07 FWD, and ermB pJB07 REV. If plasmids were still present after overnight growth, the culture was passaged again via 100-fold dilution in BHIS broth with 2% xylose, grown overnight at 37°C, and screened once more via the above method. All mutants were continually passaged until colonies successfully cured of both plasmids were obtained. Construction of the MAJ4 strain was performed by deleting *yqeC* from an isolate of MAJ2 containing pJB06 using the same methodology. Specifically, MAJ2 pJB06 was cured of pMJ18, conjugated with pMJ21, and plated on xylose. The resulting mutation was confirmed via colony PCR, and both plasmids were cured as described above.

## SUPPLEMENTAL MATERIAL

Supplemental figures and tables spectrum.00844-24-s0001.pdf Fig. S1-S5; Tables S1 and S2.

ASM does not own the copyrights to Supplemental Material that may be linked to, or accessed through, an article. The authors have granted ASM a non-exclusive, world-wide license to publish the Supplemental Material files. Please contact the corresponding author directly for reuse.
