# Supplementary material for: Clostridioides difficile exploits xanthine and uric acid as nutrients by utilizing a selenium-dependent catabolic pathway
Source: Microbiol Spectr. 2024 Aug 21;12(10):e00844-24. doi: 10.1128/spectrum.00844-24 (PMC11448449; doi:10.1128/spectrum.00844-24)
Supplement: Supplemental figures and tables — Fig. S1-S5; Tables S1 and S2. [file spectrum.00844-24-s0001.pdf]

Supplemental Information

Table S1. Genome IDs and locations for genes encoding putative selenium-dependent molybdenum hydroxylases in *C. difficile* 630 and R20291.

Table S2. Oligonucleotides used in this study.

Figure S1. *C. difficile* contains five gene clusters putatively encoding molybdenum hydroxylases.

Figure S2. Addition of hypoxanthine, xanthine, or uric acid does not affect growth of R20291 and JIR8094 in BHIS and CDMM.

Figure S3. In-frame deletions of *yqeB* and *yqeC* were successfully constructed in *C. difficile* R20291.

Figure S4. Deletion of *yqeB* and *yqeC* does not affect growth of *C. difficile* in BHIS and CDMM.

Figure S5. A wild-type copy of *yqeB* provided *in trans* fully complements the Δ*yqeB* mutant and partially complements the Δ*yqeB* Δ*yqeC* mutant.

Table S1. Genome IDs and locations for genes encoding putative selenium-dependent molybdenum hydroxylases in *C. difficile* 630 and R20291.

**Gene ID (630, R20291)**	**Gene name (*E. coli* designation)**	**Genome Location (630)**	**Genome Location (R20291)**
CD630_20730, CDR20291_1980	*xdhA1*	2,373,538..2,375,844	2,309,694..2,312,000
CD630_20790, CDR20291_1986	*xdhA2*	2,383,198..2,385,489	2,319,354..2,321,645
CD630_20870, CDR20291_1994	*xdhA3*	2,393,402..2,395,618	2,329,558..2,331,774
CD630_20990, CDR20291_2006	*xdhA4*	2,408,599..2,410,863	2,344,756..2,347,020
CD630_31770, CDR20291_3033	*xdhA5*	3,698,060..3,700,621	3,617,878..3,620,439
CD630_20740, CDR20291_1981	*xdhB1*	2,375,837..2,376,631	2,311,993..2,312,802
CD630_20800, CDR20291_1987	*xdhB2*	2,385,479..2,386,273	2,321,635..2,322,459
CD630_21010, CDR20291_2008	*xdhB3*	2,411,316..2,412,104	2,347,473..2,348,261
CD630_20810, CDR20291_1988	*xdhC1*	2,386,312..2,386,758	2,322,468..2,322,935
CD630_20880, CDR20291_1995	*xdhC2*	2,395,621..2,396,091	2,331,777..2,332,247
CD630_21000, CDR20291_2007	*xdhC3*	2,410,867..2,411,316	2,347,024..2,347,473

Table S2. Oligonucleotides used in this study.

Oligonucleotide	Description	Sequence (5'→3')
pJB07 empty FWD	To delete *pyrE* homology arms and generate empty vector for re-targeting	CTCGAGCCTGCAGACATGCA
pJB07 empty REV		GCGGCCGCGGTCATAGC
yqeB-UA-fwd	To amplify 500 bp upstream of *yqeB* for gene deletion	TTATCAGGAAACAGCTATGACCGCGGCCGCATAGATTTACTTAAGTCTAAAGGAGCTAAA
yqeB-UA-rev		TAAAATATTAATAGCTTTATCTAATATAAAGTATTACCACTCCCAATAATATTATTAATA
yqeB-DA-fwd	To amplify 500 bp downstream of *yqeB* for gene deletion	TTTATATTAGATAAAGCTATTAATATTTTATTAAGATGATATAATAAATTTAATAGATTA
yqeB-DA-rev		CAGTGCCAAGTTGCATGTCTGCAGGCTCGAGCTAATACCATTATGAGCAGCTTGTATTAT
yqeC-UA-fwd	To amplify 500 bp upstream of *yqeC* for gene deletion	TTATCAGGAAACAGCTATGACCGCGGCCGCGAGATAAAAGGAAAATTAGAATATCTATGT
yqeC-UA-rev		ATATAATATCATATAAACTATTTTAATACTACTTTCACCTCTTATAACCAGTTTTCTAAT
yqeC-DA-fwd	To amplify 500 bp downstream of *yqeC* for gene deletion	AGTATTAAAATAGTTTATATGATATTATATATATAGATAATATCAATAGAGATATTATAA
yqeC-DA-rev		ACGGCCAGTGCCAAGTTGCATGTCTGCAGGCTCGAGTTCCACACATATCTTTGTATCTTG
yqeB gRNA 3 RTH	To mutate *pyrE* gRNA to *yqeB* gRNA	TTAGAAGCCATACTAAACAAGTTTTAGAGCTAGAAATAGCAAGTTAAAATAAGGCTAGT
yqeC gRNA 2 RTH	To mutate *pyrE* gRNA to *yqeC* gRNA	ATAAAACTACAAAAACCATAGTTTTAGAGCTAGAAATAGCAAGTTAAAATAAGGCTAGT
pJB07 gRNA rev RTH	To be paired with any 'Round-the-horn forward primer for gRNA mutation	GGTACCCCCTCCTTGAATGCC
catP pJB06 FWD	To PCR check for presence of pJB06 in conjugation and plasmid curing experiments	GGGAACTTAGATGGTATTTGAAAAAATTGATAAAAATAGTTGG
catP pJB06 REV		CCTGAAGTTAACTATTTATCAATTCCTGCAATTCG
ermB pJB07 FWD	To PCR check for presence of targeting plasmid in conjugation and plasmid curing experiments	GGAGTGATTACATGAACAAAAATATAAAATATTCTCAAAACTTTTTAAC
ermB pJB07 REV		CGACTCATAGAATTATTTCCTCCCGTTAAATAATAGAT
tcdB FWD	To PCR check if selected colony in conjugation experiment is *C. difficile*	ATGAGTTTAGTTAATAGAAAACAGTTAGAAAAAATGGCAA
tcdB internal rev		ATAATTTGAATGACTCTCCACCTTTAAATTCTTCAAAG
yqeB mutation check fwd	To PCR confirm successful mutation of *yqeB*	GGGGTTCTGCTGTAGCAGCT
yqeB mutation check rev		AGTAACATACAAAGTTGCCCCATCTACA
yqeC mutation check fwd	To PCR confirm successful mutation of *yqeC*	TGGAGAGAAAAATATATAGAAATTTTATAAGAATCCCATTTGGA
yqeC mutation check rev		CAATTCTTCTTTCTTACTATATGGGAATATGACTGGAT
pHN149 empty fwd	To be paired with pJB07 empty REV to delete the multiple cloning site of pHN149 and generate an empty vector for *yqeB* complementation	CTCGAGGCCTGCAGACATGC
yqeB comp fwd	To amplify *yqeB* plus 300 bp upstream and 100 bp downstream for complementation	ACAGCTATGACCGCGGCCGCAAAATAAATAACTATAAATATTTATTAGGGACTTTCTTAT
yqeB comp rev		GCATGTCTGCAGGCCTCGAGTTTACCATTTAAATTTTAAATACCCGTCATATG

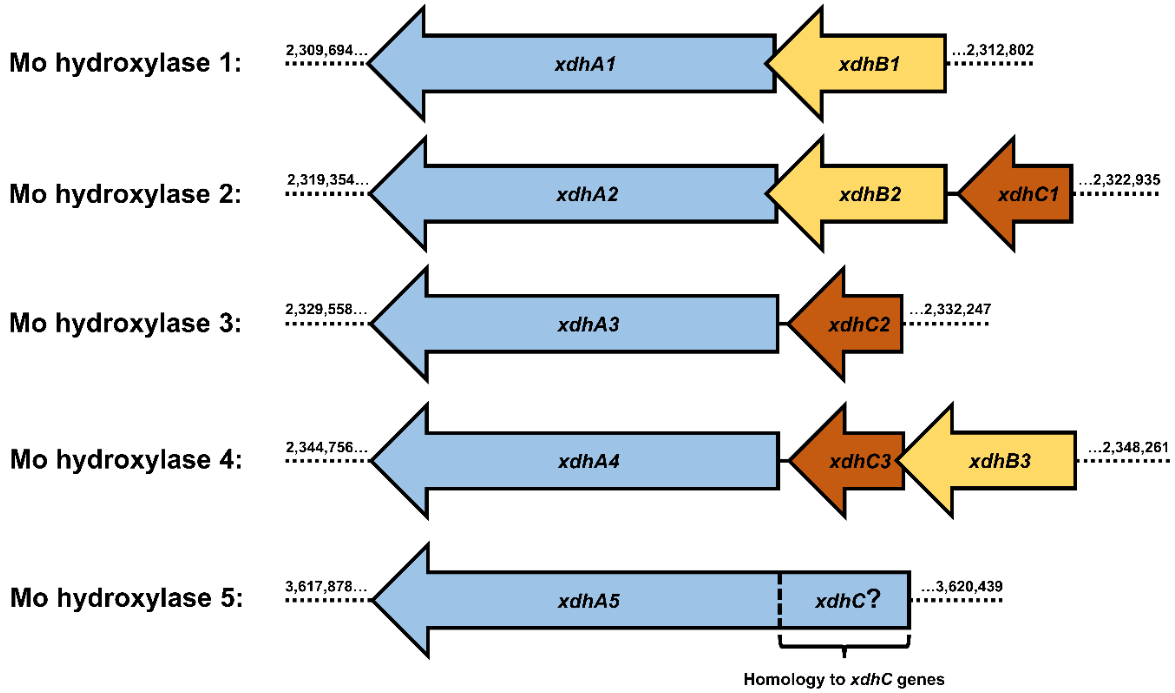

Fig. S1. *C. difficile* contains five gene clusters putatively encoding molybdenum hydroxylases. Five loci containing genes encoding putative subunits of molybdenum hydroxylases were located in the R20291 genome via tblastn of *E. faecalis* V583 EF2570. The molybdenum cofactor-binding subunits (*xdhA*), FAD-binding subunits (*xdhB*), and FeS-containing subunits (*xdhC*) are annotated using the *E. coli* nomenclature (1). *xdhA5* is isolated from the other four loci and seems to encode a molybdenum hydroxylase with a FeS-containing domain directly fused to the molybdenum center. Numbers flanking each locus indicate the location in the R20291 genome.

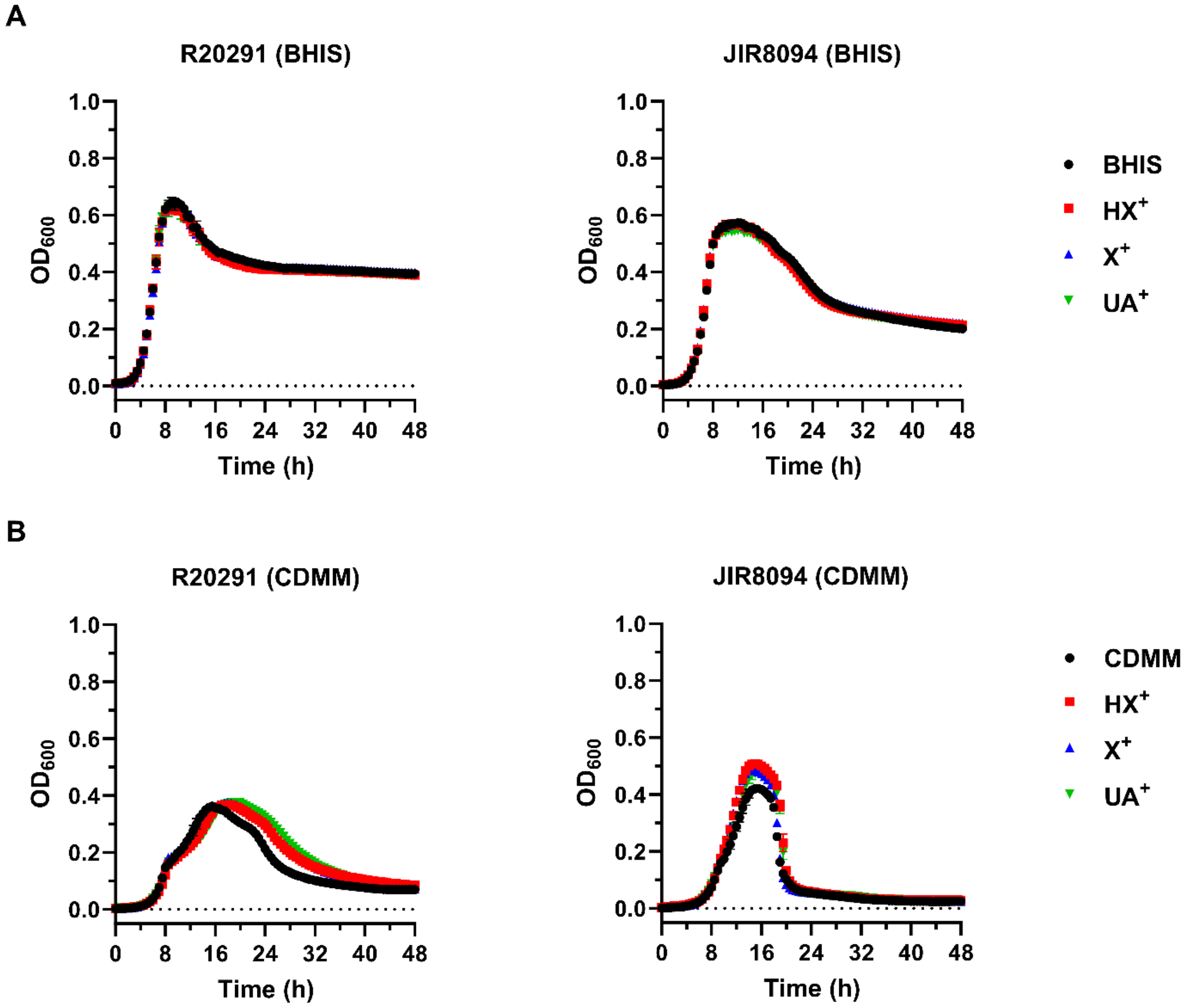

Fig. S2. Addition of hypoxanthine, xanthine, or uric acid does not affect growth of R20291 and JIR8094 in BHIS and CDMM. *C. difficile* wild-type strains R20291 and JIR8094 were grown in either (A) BHIS or (B) CDMM at 37 °C for 48 h. The turbidity (OD_{600}) of each culture was recorded every 0.5 h over the 48-h period. When indicated, hypoxanthine (HX^+), xanthine (X^+), or uric acid (UA^+) were added to each medium at 1 mM. The experiment was repeated twice. Data points represent the means of triplicate cultures while error bars represent standard deviations.

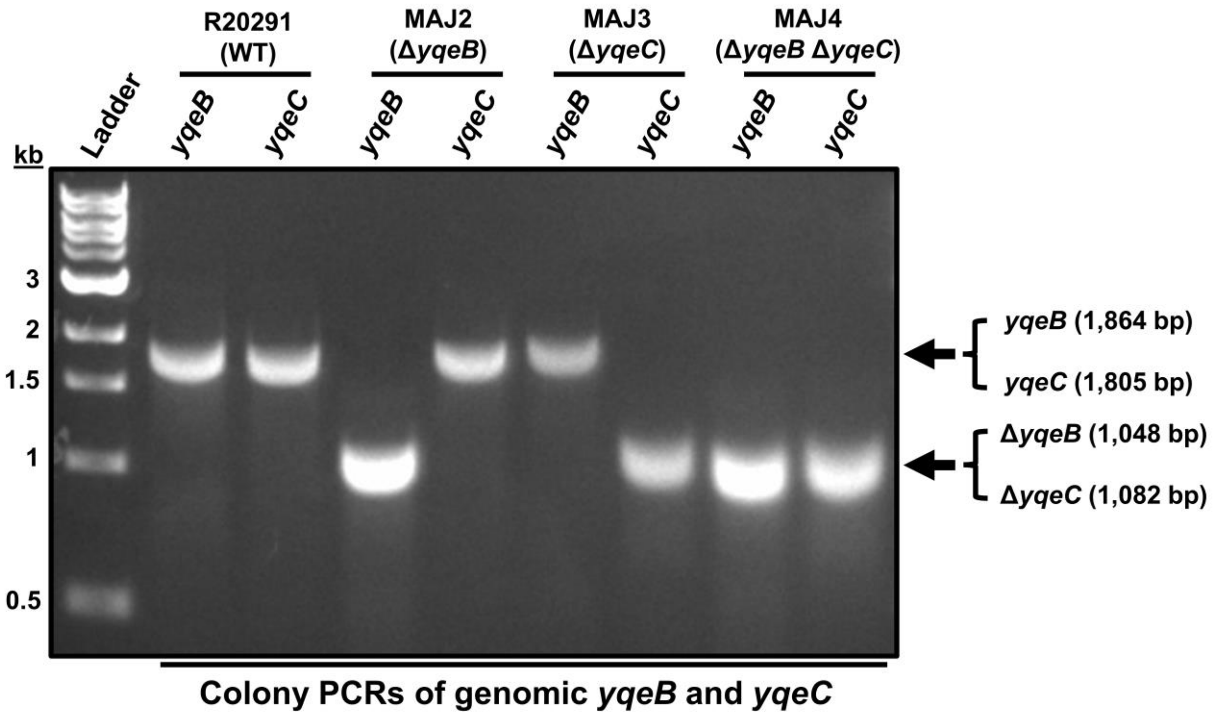

Fig. S3. In-frame deletions of *yqeB* and *yqeC* were successfully constructed in *C. difficile* R20291. *yqeB* and *yqeC* were targeted and knocked-out using a dual-plasmid xylose-inducible CRISPR-Cas9 system (2). Genomic DNA of mutant isolates plated on xylose were used in colony PCRs with primers flanking the mutation sites. R20291 was used as a control. PCR products were visualized on 1% (w/v) agarose gels containing 0.5 μg/mL ethidium bromide. Amplification of full-length *yqeB* and *yqeC* generated slower-migrating PCR products while deleted genes gave faster-migrating PCR products.

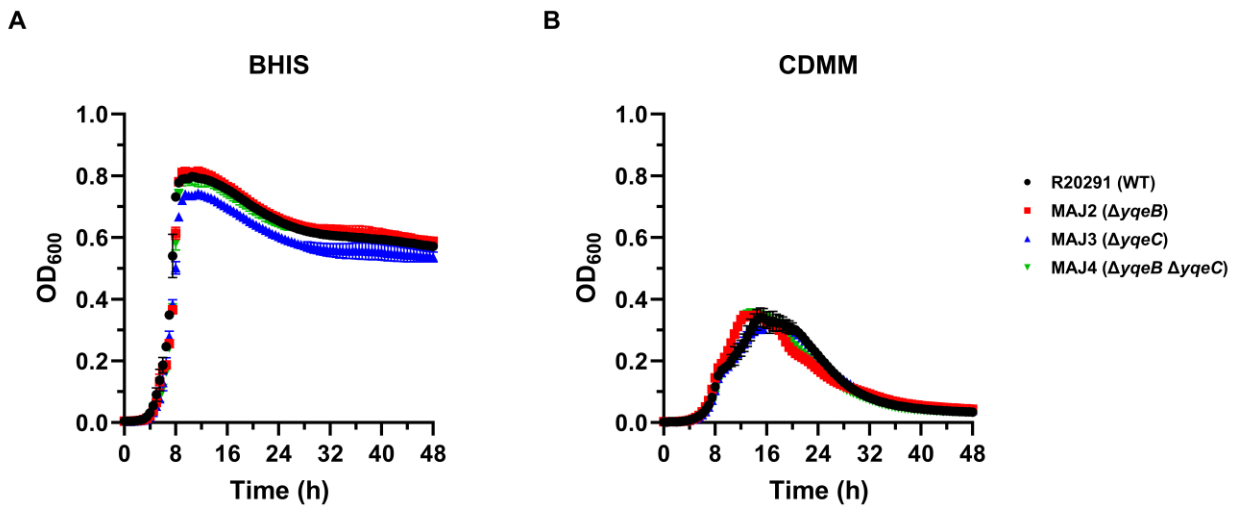

Fig. S4. Deletion of *yqeB* and *yqeC* does not affect growth of *C. difficile* in BHIS and CDMM. *C. difficile* strains R20291, MAJ2, MAJ3, and MAJ4 were grown in either (A) BHIS or (B) CDMM at 37 °C for 48 h. The turbidity (OD_{600}) of each culture was recorded every 0.5 h over the 48-h period. The experiment was repeated twice. Data points represent the means of triplicate cultures while error bars represent standard deviations.

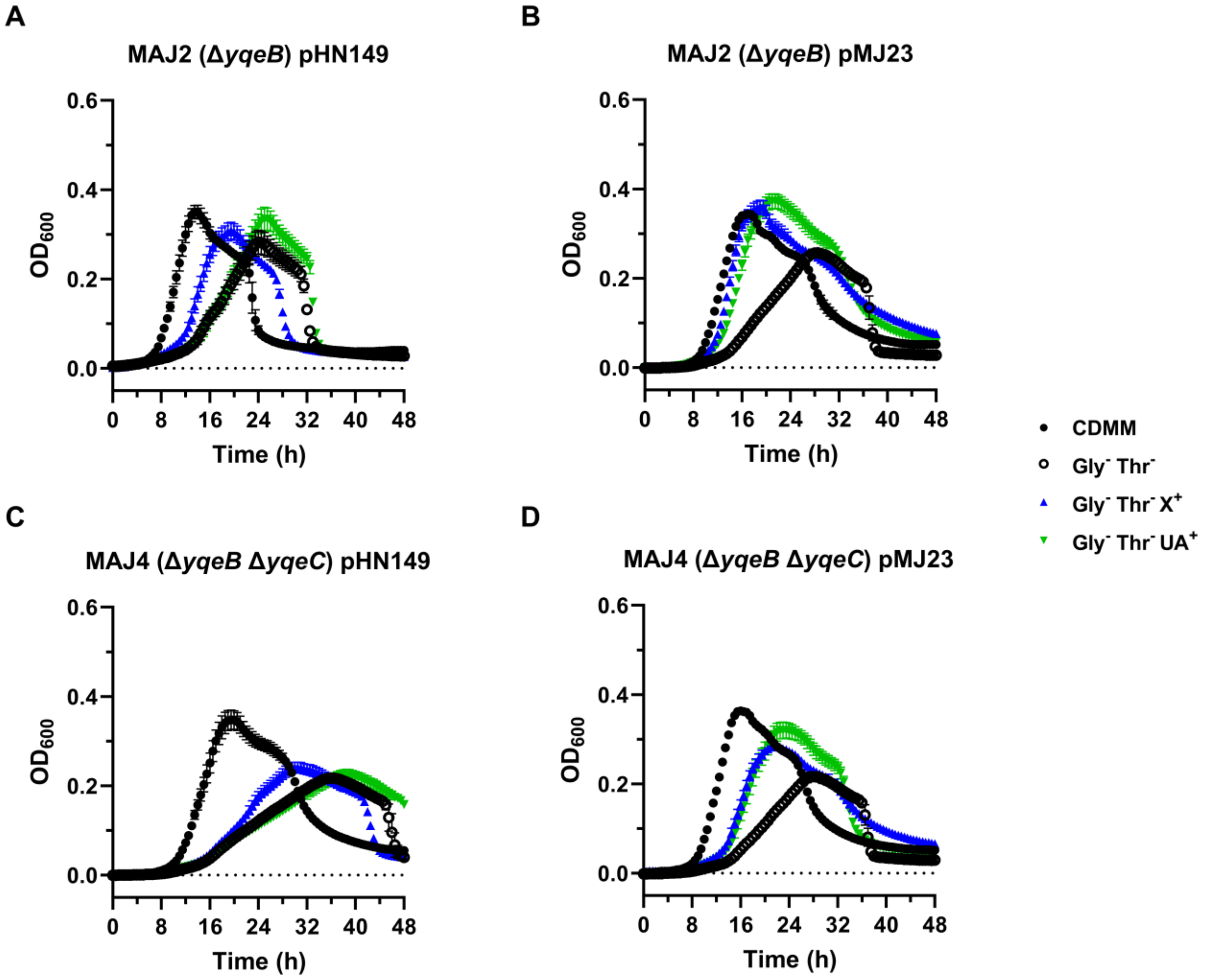

Fig. S5. A wild-type copy of *yqeB* provided *in trans* fully complements the Δ*yqeB* mutant and partially complements the Δ*yqeB* Δ*yqeC* mutant. *C. difficile* mutant strains (A, B) MAJ2 and (C, D) MAJ4 harboring either an empty shuttle vector (pHN149) or the vector containing a wild-type copy of *yqeB* (pMJ23) were grown in CDMM augmented with 10 µg/mL thiamphenicol at 37 °C for 48 h. The turbidity (OD_{600}) of each culture was recorded every 0.5 h over the 48-h period. When indicated, glycine and threonine were omitted (Gly^{-} Thr^{-}) and substituted with either 1 mM xanthine (X^{+}) or uric acid (UA^{+}). The experiment was repeated twice. Data points represent the means of triplicate cultures while error bars represent standard deviations.

REFERENCES

1. Xi H, Schneider BL, Reitzer L. 2000. Purine catabolism in *Escherichia coli* and function of xanthine dehydrogenase in purine salvage. *J Bacteriol* 182:5332-41.
2. Brehm JN, Sorg JA. 2022. Plasmid Sequence and Availability for an Improved *Clostridioides difficile* CRISPR-Cas9 Mutagenesis System. *Microbiol Resour Announc* 11:e0083322.
